# Efficient travelling-mode quantum key agreement against participant’s attacks

**DOI:** 10.1038/s41598-019-51987-z

**Published:** 2019-11-11

**Authors:** Wei-cong Huang, Yong-kai Yang, Dong Jiang, Li-jun Chen

**Affiliations:** State Key Laboratory for Novel Software Technology, Nanjing University, Nanjing, 210046 P. R. China

**Keywords:** Quantum information, Theoretical physics

## Abstract

Quantum key agreement (QKA) is to negotiate a final key among several participants fairly and securely. In this paper, we show that some existing travelling-mode multiparty QKA protocols are vulnerable to internal participant’s attacks. Dishonest participants can exploit a favorable geographical location or collude with other participants to predetermine the final keys without being discovered. To resist such attacks, we propose a new travelling-mode multiparty QKA protocol based on non-orthogonal Bell states. Theoretical analysis shows that the proposed protocol is secure against both external and internal attacks, and can achieve higher efficiency compared with existing travelling-mode multiparty QKA protocols. Finally we design an optical platform for each participant, and show that our proposed protocol is feasible with current technologies.

## Introduction

In 1984, the first quantum cryptographic protocol, known as BB84 quantum key distribution protocol was proposed by Bennett and Brassard^1^. Since its unconditional security is guaranteed by the laws of quantum mechanics, quantum cryptography has become a heated topic, and various protocols including quantum key distribution (QKD)^1,2^, quantum secure direct communication (QSDC)^3–9^, quantum secret sharing (QSS)^10,11^, etc., have been proposed. Recently, Quantum key agreement(QKA), a new branch of quantum cryptography, has attracted extensive attention.

Different from QKD, QKA can fairly and securely negotiate a final key among users. That is, the final key is equally determined by each participant and any non-trivial subset of the participants cannot absolutely predetermine the final key. In 2004, Zhou *et al*. proposed the first QKA protocol by utilizing the quantum teleportation technique^12^. In the same year, Hsueh and Chen proposed another QKA protocol by employing the entangled states^13^. Nevertheless, Tsai *et al*. pointed that neither of the two protocols is secure^14,15^. In 2010, Chong and Hwang devised a QKA protocol based on BB84^16^. However, the above protocols are all based on two-party. To extend QKA to the multi-party case, Shi and Zhong designed the first multiparty QKA (MQKA) protocol based on Bell states in 2013^17^. Since then, many MQKA protocols using single or entanglement quantum states have been proposed^18–34^.

Liu^18^ pointed out that existing MQKA protocols can be classified into three types according to the transmission topology of quantum photons: complete-graph-type^17,20^, circle-type^19,21–34^ (also known as travelling-mode) and tree-type^35^. In the first type, every participant sends each of other participants a sequence of photons which carries the information of his/her secret key. In the second type, each participant only sends out one sequence, which will be operated by each of other participants by turns and sent back to the one who prepares it. The third type is one participant generates a sequence of high dimensional photon states (e.g. GHZ states) and sends each of other participants one of its particles. Since the travelling-mode is more efficient than complete-graph-type and easier to satisfy the fairness property compared with the tree-type, it has attracted comprehensive study. In 2013, Sun *et al*. presented a MQKA protocol^19^ in travelling-mode to improve the efficiency of Liu *et al*’s MQKA protocol^20^. In 2014, Shukla *et al*. proposed a travelling-mode MQKA protocol based on Bell state and Bell measurements^21^. In 2015, Zhu *et al*. put forward the attack strategy to defeat Shukla *et al*’s protocol and proposed an improved version^22^. In 2018, Abulkasim pointed out that Wang and Ma’s protocol^23^ is susceptible to participant’s attacks and proposed an improved protocol^24^. Meanwhile, Cao and Ma proposed two MQKA protocols which were designed to be immune to the collusive attack^25^; they also presented a MQKA protocol based on non-orthogonal quantum entangled pairs^34^. Besides, some protocols based on higher-dimensional quantum states, such as five-qubit brown states^26^, G-Like states^28^, and four-qubit symmetric W state^29^, were presented.

In these travelling-mode MQKA protocols, we find that some protocols^25–30^ cannot resist dishonest participant’s attacks, which leads to the failure of fairness property^19^. The dishonest participant can take advantage of a favorable geographical location or collude with other participants to predetermine the final keys of honest participants without being discovered. Besides, we also find there exists the problem of information leakage in Cao-Ma MQKA protocol^34^. Following we take two Cao-Ma MQKA protocols^25,34^ as examples to demonstrate the attacks in detail. To resist these attacks, We propose a new MQKA protocol based on non-orthogonal Bell states by utilizing Pauli and rotation operations. Our proposed protocol has three noticeable advantages: Firstly, owing to the use of non-orthogonal Bell states, the proposed protocol can resist attacks from both internal dishonest participants and external eavesdroppers. It also effectively solves the problem of information leakage in Cao-Ma protocol. Secondly, the frequency of eavesdropping detection has been greatly reduced. Hence, the qubit efficiency and measurement efficiency of our proposed protocol are higher than those of the existing secure ones^20,32–34^. Thirdly, since only Bell states and unitary operations are employed, the protocol is feasible with the current technology.

The rest of the paper is organized as follows. Next section first reviews and analyzes the security of Cao-Ma MQKA protocols, then introduces our improved travelling-mode MQKA protocol in detail, followed by the security analysis and efficiency comparisons with existing secure protocols. Furthermore, an optical setup is provided. Finally, a short conclusion of this paper is given in the final section.

## Results

### Review of Cao-Ma MQKA protocols

In this section we briefly describe the Cao-Ma MQKA protocol 1^25^ without trust party and Cao-Ma MQKA protocol 2^34^ based on non-orthogonal quantum entangled pairs respectively.

#### Cao-Ma MQKA protocol 1

The main process of Cao-Ma MQKA protocol without trust party can be divided into two stages. The first stage is initialization and encoding stage. Each participant *P*_*i*_(*i* = 0, 1, …, *N*−1) possesses a *n*-bit 0–1 sequence ${\tilde{K}}_{i}$ and *TS*_*i*_ as his secret key and additional random sequence, and calculates ${K}_{i}={\tilde{K}}_{i}\oplus T{S}_{i}$. Then he prepares a sequence of Bell states randomly selected from four Bell states, wherein the states of the photon sequence can be expressed as *W*_*i*_. Each participant keeps the first photon sequence in his hand and sends the second photon sequence which is inserted into decoy photons to next participant *P*_*i*+1_. *P*_*i*_ and *P*_*i*+1_ perform eavesdropping checking. If the communication is secure, *P*_*i*+1_ performs one of the four Pauli operations on the received photon sequence according to *K*_*i*+1_. Next, *P*_*i*+1_ inserts decoy photons into the photon sequence and sends it to next participant *P*_*i*+2_. This process continues until *P*_*i*_ gets the sequence which he generated. The second stage is final key negotiation stage. After each participant gets the sequence he generates, he performs Bell measurements on corresponding photon pairs. The measurement results of the sequence can be expressed as *V*_*i*_. Then each participant *P*_*i*_ publishes his random sequence *TS*_*i*_ and calculates TS = *TS*_0_ ⊕ *TS*_1_ ⊕ … ⊕ *TS*_*N*−1_. Finally, each participant can obtain the final common key *K*_*c*_, where ${K}_{c}=TS\oplus {W}_{i}\oplus {V}_{i}\oplus {\tilde{K}}_{i}$.

#### Cao-Ma MQKA protocol 2

This protocol is based on non-orthogonal quantum pairs and adopts the idea of Pauli and Hadamard operations mixed encoding. The process is as follows. Firstly, there are eight photon pairs which are in *BS* = {|*ϕ*^+^〉, |*ϕ*^−^〉, |*ψ*^+^〉, |*ψ*^−^〉} and *DBS* = {*H*|*ϕ*^−^〉, *H*|*ϕ*^+^〉, *H*|*ψ*^−^〉, *H*|*ψ*^+^〉}, and four unitary operations in *U* = {*U*_00_, *U*_11_, *U*_01_, *U*_10_} = {*I*, *iY*, *H*, *iYH*}, wherein *H* is Hadamard operation. Each participant *P*_*i*_(*i* = 0, 1, …, *N*−1) generates a sequence of classical bits *K*_*i*_ as his private key, wherein *K*_*i*_ ∈ {00, 11, 01, 10}. Moreover, *P*_*i*_ also generates a sequence *C*_*i*_, where *C*_*i*_ is 0 if *K*_*i*_ ∈ {00, 11} and *C*_*i*_ is 1 if *K*_*i*_ ∈ {01, 10}. Then each participant *P*_*i*_ prepares a random quantum pair sequence from *BS* or *DBS* and transmitted the second photon sequence to the next participant *P*_*i*+1_. After receiving the sequence, *P*_*i*+1_ executes the eavesdropping checking and performs unitary operations on the received quantum sequence according to his private key. Until each participant has encoded his private key on the photon sequences of others and receives the sequence he generates, he publishes a classical sequence *C*_*i*_ to reveal the measurement basis and calculates *C* = *C*_1_ ⊕ *C*_2_ ⊕ … ⊕ *C*_*N*_. Each participant performs *BS* or *DBS* measurements on the photon pairs according to *C*. If *C* is 0, the measurement basis is the same as the initial state; otherwise, the measurement basis is the dual basis of the initial states. Finally, all participants can extract the common key by comparing the initial states and measurement results.

### Security analysis of the Cao-Ma MQKA protocols

In this section, we first show that the dishonest participant in Cao-Ma MQKA protocol 1 can take advantage of a favorable geographical location or collude with other participants to predetermine the final key without being discovered, leading to the failure of fairness property. Next we reveal the problem of information leakage in Cao-Ma MQKA protocol 2.

#### Fairness analysis

In travelling-mode MQKA protocols, participants encode their secret keys on photons by performing the unitary operations. Besides, they usually perform additional random operations on photons in case to divulge the secret keys. Therefore, once the additional operation is obtained, the participant will deduce the final key directly. Following we take the tripartite (Alice, Bob and Charlie) example to introduce the attack strategy. Suppose Bob is a dishonest participant, his detailed attack process is as follows.

1. Before Alice and Bob publish the random sequence *TS*_*A*_ and *TS*_*C*_, Bob selects an advantageous geographical position aside Alice and Charlie so that he can get *TS*_*A*_ and *TS*_*C*_ earlier than expected.
2. Once Bob gets the sequence, he calculates the final key $K=T{S}_{A}\oplus T{S}_{C}\oplus {\tilde{K}}_{B}\oplus {W}_{B}\oplus {V}_{B}$ and *M* = *K* ⊕ *K*′, where *K*′ is the final key he excepts.
3. Then Bob informs Alice and Charlie of $T{S^{\prime} }_{B}$ = *M* ⊕ *TS*_*B*_. Thus, Alice and Charlie will get the illegal final keys *K*_*A*_ = ${\tilde{K}}_{A}\oplus T{S^{\prime} }_{B}\oplus T{S}_{C}\oplus {W}_{A}\oplus {V}_{A}$ = *K*′ and *K*_*C*_ = ${\tilde{K}}_{C}\oplus T{S^{\prime} }_{B}\oplus T{S}_{A}\oplus {W}_{C}\oplus {V}_{C}$ = *K*′ as Bob anticipates.

Through the above operations, Bob can determine the final keys of Alice and Charlie. Following, we will analyze the collusion attack in detail. For clarity, we assume that four participants *P*_0_, *P*_1_, *P*_2_ and *P*_3_ want to generate the final key. *P*_1_ and *P*_3_ are dishonest and want to steal *P*_2_’s secret key. The detailed attack process is as follows:

1. *P*_0_ prepares a sequence of Bell states |*PS*_1_^0^*PS*_2_^0^〉, then he transmits the second photon sequence |*PS*_2_^0^〉 with decoy photons $|{\tilde{PS}}_{2}^{0\to 1}\rangle s$ to *P*_1_.
2. *P*_1_ performs unitary operations on the received photons according to his secret key and sends the sequence $|{\tilde{{U}^{1}PS}}_{2}^{0\to 1}\rangle$ to *P*_3_ instead of *P*_2_ as illustrated in Fig. 1. Meanwhile, *P*_1_ prepares a fake sequence of Bell states |*S*_1_^1^*S*_2_^1^〉 and sends the first photon sequence $|{\tilde{S}}_{1}^{1\to 3}\rangle$ to *P*_3_ and the second photon sequence $|{\tilde{S}}_{2}^{1\to 2}\rangle$ to *P*_2_.

**Figure 1:**
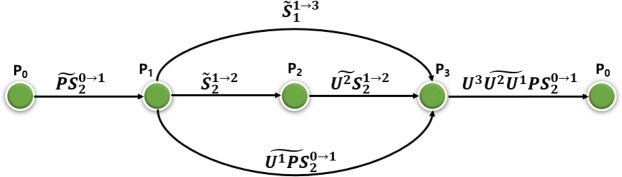
Dishonest participants’ collusive attack strategy. *P*_1_ and *P*_3_ collude to eavesdrop on the honest participant *P*_2_’s secret key.
3. After security checking, as *P*_2_ does not know the received photon sequence is fake, he encodes the sequence by performing unitary operations according to *TS*_2_ and sends the sequence $|{\tilde{{U}^{2}S}}_{2}^{1\to 2}\rangle$ to *P*_3_.
4. After confirming *P*_3_ has received the sequence $|{\tilde{{U}^{2}S}}_{2}^{1\to 2}\rangle$, *P*_2_ and *P*_3_ execute eavesdropping checking. If the communication is secure, *P*_1_ and *P*_3_ will perform Bell measurement on |*S*_1_^1→3^〉 and |*U*^2^*S*_2_^1→2^〉. Then they can get *P*_2_’s unitary operations, i.e., ${\tilde{K}}_{2}$ ⊕ *TS*, by comparing the measurement results and initial states.
5. *P*_3_ encodes photon sequence |*U*^1^*PS*_2_^0→1^〉 with *P*_2_’s unitary operations and his unitary operations. *P*_3_ also generates some decoy photons and inserts them in to |*U*^3^*U*^2^*U*^1^*PS*_2_^0→1^〉 randomly. Then he sends the sequence to *P*_0_.
6. *P*_1_ and *P*_3_ wait for the common key negotiation stage, where every participant publishes his *TS*_*i*_. By comparing *P*_2_’s unitary operations and *TS*_2_, *P*_1_ and *P*_3_ can effortlessly recover ${\tilde{K}}_{2}$. For example, suppose the fake photon pairs prepared by *P*_1_ is |*ϕ*^+^〉, and the result of Bell measurement by *P*_1_ and *P*_3_ is |*ϕ*^+^〉 after *P*_2_’s encoding, they can deduce the operation performed by *P*_2_ is *U*_00_. Assume the *TS*_2_ published by *P*_2_ is 01, *P*_1_ and *P*_3_ can definitely deduce the *P*_2_’s secret key is 01 according to Table 1. Then *P*_1_ and *P*_3_ can determine *P*_2_’s final key by announcing fake *TS*_1_ and *TS*_3_.

**Table 1:** Relationship between *P*_1_’s photon states, *P*_2_’s operations and *P*_3_’s measurement results.

Initial State	$\tilde{{K}_{2}}\oplus {\rm{TS}}$	Final State
|*ϕ*^+^〉	00 ⊕ 00	|*ϕ*^+^〉
	11 ⊕ 11	
	01 ⊕ 01	
	10 ⊕ 10	
|*ϕ*^+^〉	00 ⊕ 11	|*ϕ*^−^〉
	11 ⊕ 00	
	01 ⊕ 10	
	10 ⊕ 01	
|*ϕ*^+^〉	00 ⊕ 01	|*ψ*^+^〉
	11 ⊕ 10	
	01 ⊕ 00	
	10 ⊕ 11	
|*ϕ*^+^〉	00 ⊕ 10	|*ψ*^−^〉
	11 ⊕ 01	
	01 ⊕ 11	
	10 ⊕ 00

In addition to the Cao-Ma protocol 1, these agreements^26–30^ are also vulnerable to dishonest participants’ collusion attack, where indicates the protocols cannot satisfy the fairness property.

#### Information leakage analysis

Information leakage is that Eve can extract some information about secret key without any active attack^36^. In Cao-Ma MQKA protocol 2, each participant *P*_*i*_ needs to publish a classical sequence *C*_*i*_ after he receives the sequence he generates. However, *C*_*i*_ and *K*_*i*_ are closely related. If *C*_*i*_ is 0, Eve can draw a conclusion that the secret key of *P*_*i*_ must be 00 or 11; otherwise, the *K*_*i*_ is 01 or 10, which contains $-2\times \frac{1}{2}\,lo{g}_{2}\frac{1}{2}=1$ bit of information. Thus, one bit of the secret information is leaked to Eve unconsciously.

### The improved travelling-mode MQKA protocol

Herein we design a new travelling-mode MQKA protocol based on non-orthogonal Bell states, where *n* participants negotiate a final key fairly and securely. The detailed process of our protocol is as follows:

#### Initialization phase

Each participant *P*_*i*_ first generates a (*l* + *kl*)-bit 0–1 secret key sequence *K*_*i*_ = {*K*_*i*,1_, *K*_*i*,2_, …, *K*_*i*,*m*_}, *m* ∈ {1, 2, …, *l* + *kl*}. Besides he also generates a random (*l* + *kl*)-bit 0–1 controlling string *RH*_*i*_^*j*^, where *k* is the detection rate, i, j ∈ {1, 2, …, *n*} and i ≠ j. Then *P*_*i*_ prepares a sequence *BS*_*i*_ = {|*BS*_*wi*,1 *wi*,2_^*i*^〉, |*BS*_*wi*,3 *wi*,4_^*i*^〉, …, |*BS*_*wi*,2(*l*+*kl*)−1 *wi*,2(*l*+*kl*)_^*i*^〉} of *l* + *kl* Bell states, where |*BS*_*wi*,2*m*−1 *wi*,2*m*_^*i*^〉 ∈ {|*BS*_00_〉, |*BS*_01_〉, |*BS*_10_〉, |*BS*_11_〉} and *W*_*i*_ = (*w*_*i*,1_, *w*_*i*,2_, …, *w*_*i*,2(*l*+*kl*)−1_, *w*_*i*,2(*l*+*kl*)_) is a random 2(*l* + *kl*)-bit 0–1 sequence.

1 $$\begin{array}{l}|B{S}_{00}\rangle =|{{\varphi }}^{+}\rangle =\frac{1}{\sqrt{2}}\mathrm{(|00}\rangle +\mathrm{|11}\rangle ),\,|B{S}_{01}\rangle =|{{\varphi }}^{-}\rangle =\frac{1}{\sqrt{2}}\mathrm{(|00}\rangle -\mathrm{|11}\rangle ),\\ |B{S}_{10}\rangle =|{\psi }^{+}\rangle =\frac{1}{\sqrt{2}}\mathrm{(|01}\rangle +|10\rangle ),\,|B{S}_{11}\rangle =|{\psi }^{-}\rangle =\frac{1}{\sqrt{2}}\mathrm{(|01}\rangle -\mathrm{|10}\rangle \mathrm{).}\end{array}$$

#### Sending photons

*P*_*i*_ divides *BS*_*i*_ into two single photon sequence: the first photon sequence |*BS*_1_^*i*→*i*^〉 and the second photon sequence |*BS*_2_^*i*→*i*+1^〉 (symbol ‘+’ in *i* + 1 denotes the additional mod *n*). Then *P*_*i*_ keeps the first photon sequence in home and transmits the second photon sequence to the next participant *P*_*i*+1_.

#### Encoding phase

After *P*_*i*+1_ receives the photon sequence, he performs unitary operation *I* or *Z* on |*BS*_2_^*i*→*i*+1^〉 according to his private key sequence, where *I* = |0〉〈0| + |1〉〈1| and *Z* = |0〉〈0| − |1〉〈1|.

#### Controlling operations

Depending on whether the sequence *RH*_*i*+1_^*i*^ is 1 or 0, *P*_*i*+1_ performs rotation operation ${R}_{z}(\frac{\pi }{2})$ on the sequence |*BS*_2_^*i*→*i*+1^〉 or does nothing, where ${R}_{z}(\frac{\pi }{2})$ is the rotation operator of the z axis and the definition is as follows:

2 $${R}_{z}(\frac{\pi }{2})=\frac{\sqrt{2}}{2}I-\frac{\sqrt{2}}{2}iZ=[\begin{array}{cc}\frac{\sqrt{2}}{2}-\frac{\sqrt{2}}{2}i & 0\\ 0 & \frac{\sqrt{2}}{2}+\frac{\sqrt{2}}{2}i\end{array}]$$

The rotation operator can change the state of the |*BS*〉 to |*DBS*〉 = {|*DBS*_00_〉, |*DBS*_01_〉, |*DBS*_10_〉, |*DBS*_11_〉}, where |*DBS*〉 are defined as follows. Table 2 shows the relationship of the unitary operations and the transformed Bell states.

3 $$\begin{array}{c}|DB{S}_{00}\rangle ={R}_{z}(\frac{\pi }{2})|{{\varphi }}^{+}\rangle =\frac{1}{\sqrt{2}}(|{{\varphi }}^{+}\rangle -i|{{\varphi }}^{-}\rangle ),\\ |DB{S}_{01}\rangle ={R}_{z}(\frac{\pi }{2})|{{\varphi }}^{-}\rangle =\frac{1}{\sqrt{2}}(|{{\varphi }}^{-}\rangle -i|{{\varphi }}^{+}\rangle ),\\ |DB{S}_{10}\rangle ={R}_{z}(\frac{\pi }{2})|{\psi }^{+}\rangle =\frac{1}{\sqrt{2}}(|{\psi }^{+}\rangle +i|{\psi }^{-}\rangle ),\\ |DB{S}_{11}\rangle ={R}_{z}(\frac{\pi }{2})|{\psi }^{-}\rangle =\frac{1}{\sqrt{2}}(|{\psi }^{-}\rangle +i|{\psi }^{+}\rangle \mathrm{).}\end{array}$$

**Table 2:** Effects of unitary operations {*I*, *Z*, *R*_*z*_*I*, *R*_*z*_*Z*} on the second particles of Bell states |BS〉.

State	|*BS*_00_〉	|*BS*_01_〉	|*BS*_10_〉	|*BS*_11_〉
Operation				
*I*	|*BS*_00_〉	|*BS*_01_〉	|*BS*_10_〉	|*BS*_11_〉
*Z*	|*BS*_01_〉	|*BS*_00_〉	|*BS*_11_〉	|*BS*_10_〉
*R*_*z*_ *I*	|*DBS*_00_〉	|*DBS*_01_〉	|*DBS*_10_〉	|*DBS*_11_〉
*R*_*z*_ *Z*	|*DBS*_01_〉	|*DBS*_00_〉	|*DBS*_11_〉	|*DBS*_10_〉

After performing the extra unitary operation, *P*_*i*+1_ sends the sequence |*BS*_2_^*i*→*i*+2^〉 to the next participant *P*_*i*+2_. Meanwhile, each of the other *n* − 1 participants processes his received sequence just in the same way and sends the obtained new sequence to next participant. This process continues until *P*_*i*_ receives the sequence which he generated from *P*_*i*−1_.

#### Security checking

Once receiving his own sequence, each participant *P*_*i*_ announces the fact, confirms other participants have received their sequences and informs participant *j* of his controlling sequence *RH*_*i*_^*j*^. Then all participants cooperate to choose *kl* positions from *l* + *kl* Bell states for security checking, and the remaining *l* Bell states are used to form the final key, i.e., *K*_*i*_^′^, i= 1, 2, 3, …, n. Specifically, *P*_*i*_ randomly selects $\frac{kl}{n}$ positions from the remaining $(l+kl)-(i-\mathrm{1)}\frac{kl}{n}$ positions and announces the positional information. After finishing selection, each participant publishes the secret key sequence at the *kl* positions. Then they calculate the *XOR* results of other participants’ secret keys, offset the extra controlling operations according to *RH*_*j*_^*i*^ (the detailed process is shown in the next step) and perform Bell measurements on the photon pairs at their chosen $\frac{kl}{n}$ positions. If the measurements are consistent with the calculations, they drop the *kl* bits used for security checking and continue; otherwise they terminate the protocol.

#### Secret extraction

Each participant *P*_*i*_ offsets the controlling operations for the remaining *l* positions according to *RH*_*j*_^*i*^. Concretely, since ${R}_{z}(\frac{\pi }{2})$ commutes with each of the encoding operations {*I*, *Z*}, we can deduce that ${R}_{z}(\frac{\pi }{2})I=I{R}_{z}(\frac{\pi }{2})$ and ${R}_{z}(\frac{\pi }{2})Z=Z{R}_{z}(\frac{\pi }{2})$. Therefore, each participant *P*_*i*_ can offset all controlling operations ${R}_{z}(\frac{\pi }{2})$ by repeating *C*_*i*_^*j*^ operations ${R}_{z}{(\frac{\pi }{2})}^{\dagger }$ (i.e., by performing once operation ${R}_{z}(\frac{-{C}_{i}^{j}\pi }{2})$) on the j-th pair of photons in sequence *BS*_*i*_ after all the participants have completed their encoding operations *E* and controlling operations *C* in turn: *CECECE* = *CCCEEE*, where *C*_*i*_^*j*^ is the j-th bit of the sequence *C*_*i*_ (*C*_*i*_ = *RH*_*i*+1_^*i*^ + *RH*_*i*+2_^*i*^ + … + *RH*_*i*−1_^*i*^), ${R}_{z}{(\frac{\pi }{2})}^{\dagger }={({R}_{z}{(\frac{\pi }{2})}^{{\rm T}})}^{\ast }$ and ${R}_{z}{(\frac{\pi }{2})}^{\dagger }{R}_{z}(\frac{\pi }{2})=I$. After that, each participant performs Bell measurements on the *l* processed photon pairs and obtains $\bar{{K{}^{{\rm{^{\prime} }}}}_{i}}={K{}^{{\rm{^{\prime} }}}}_{i+1}\oplus {K{}^{{\rm{^{\prime} }}}}_{i+2}\oplus \ldots \oplus {K{}^{{\rm{^{\prime} }}}}_{i-1}$. Finally *P*_*i*_ can get the final key $K=K{{}^{{\rm{^{\prime} }}}}_{i}\oplus {\bar{K{}^{{\rm{^{\prime} }}}}}_{i}$.

So far, we have demonstrated our proposed travelling-mode MQKA protocol. In the real scenario, the raw keys may have very few mistakes which are caused by the channel noise. We can use the multiparty cascade error-correcting protocols for information reconciliation^37,38^ and utilize the universal hashing to realize privacy amplification process^39^.

### Security analysis

Herein we give a detailed security analysis for both outside and participant’s attacks. It is proved that the proposed protocol can satisfy the fairness property effectively. We also show the problem of information leakage does not exist in our protocol.

#### Outside Attacks

Suppose Eve wants to eavesdrop the final key, he should obtain each participant’s private key first. Here are three mainstream attack methods he may take.

Firstly, let us discuss the intercept-resend attack^25,35^. In intercept-resend attack, Eve intercepts and stores the photon sequences sent from participant *P*_*i*_ to *P*_*i*+1_. Then he sends the second photon sequence of the fake Bell states which he prepared in advance to *P*_*i*+1_. After step (3) and (4), *P*_*i*+1_ finishes performing his unitary operations and extra controlling operations on the photon sequence and sends to *P*_*i*+2_. At this time Eve will intercept the photon sequence again and sends the original photon sequence to *P*_*i*+2_. Since Eve does not know whether *P*_*i*+1_ performs the controlling operation ${R}_{z}(\frac{\pi }{2})$ on the photons or not, he won’t perform Bell measurements on his photon sequence until each participant publishes the random controlling sequence. Therefore he cannot deduce *P*_*i*+1_’s operations and encode correct information on the original sequence. Eve will be detected with the probability $1-{(\frac{1}{2})}^{kl}\approx 1$ (*kl* is big enough) when all participants perform security checking in step (5). Hence the proposed protocol can resist the intercept-resend attack.

Secondly, let us discuss the entangle-measure attack^35,40^. In entangle-measure attack, Eve wants to steal *P*_*i*+1_’s secret key by intercepting the traveling photon sequence |*BS*_2_^*i*→*i*+1^〉 and |*BS*_2_^*i*→*i*+2^〉, and executing Controlled-not operation on it and his auxiliary photon |0〉_*e*_, where intercepted photon is a control bit and photon |0〉_*e*_ is a target bit. For instance, the Bell state prepared by *P*_*i*_ is $|{\Psi }_{1}{\rangle }_{pq}=\frac{1}{\sqrt{2}}\mathrm{|00}\rangle +\mathrm{|11}\rangle s$. After Eve’s operation on *q* and *e*, the entangled state will transform to $|{\Psi }_{2}{\rangle }_{pqe}=\frac{1}{\sqrt{2}}\mathrm{|000}\rangle +\mathrm{|111}\rangle$, which is composed of three entangled particles. Then Eve sends the particle *q* to *P*_*i*+1_. After *P*_*i*+1_ performs unitary operations on the sequence and sends to *P*_*i*+2_, Eve intercepts the particle *q*, performs Controlled-not operation on *q* and *e* again and sends *q* to *P*_*i*+2_. After all participants have received their sequences, they start to announce the controlling sequence *RH*_*i*_^*j*^ and offset the extra controlling operations on the checking photons. The states can be defined as follows:

4 $$\begin{array}{rcl}{U}_{CNOT}(q\otimes e){I}_{q}|{\Psi }_{2}{\rangle }_{pqe} & = & |{{\varphi }}^{+}{\rangle }_{pq}\mathrm{|0}{\rangle }_{e},\\ {U}_{CNOT}(q\otimes e){Z}_{q}|{\Psi }_{2}{\rangle }_{pqe} & = & |{{\varphi }}^{-}{\rangle }_{pq}\mathrm{|0}{\rangle }_{e},\\ {R}_{q}^{\dagger }{U}_{CNOT}(q\otimes e){R}_{q}{I}_{q}|{\Psi }_{2}{\rangle }_{pqe} & = & |{{\varphi }}^{+}{\rangle }_{pq}\mathrm{|0}{\rangle }_{e},\\ {R}_{q}^{\dagger }{U}_{CNOT}(q\otimes e){R}_{q}{Z}_{q}|{\Psi }_{2}{\rangle }_{pqe} & = & |{{\varphi }}^{-}{\rangle }_{pq}\mathrm{|0}{\rangle }_{e}\mathrm{.}\end{array}$$

According to the Eq. (4), the state of auxiliary photon *e* is always |0〉_*e*_ whether *P*_*i*+1_’s operation is *I*, *Z*, *R*_*z*_*I* or *R*_*z*_*Z*. Therefore Eve cannot obtain *P*_*i*+1_’s secret key even if the photon *e* is entangled with transmitted photons sequence. We can consider that the Entangle-Measure attack is inefficient.

Thirdly, let us discuss the trojan horse attack. The trojan horse attack is another common attack in travelling-mode MQKA protocols which have been discussed in Li *et al*’s protocol^41^. To prevent this type of attack, participant can install some special quantum optical devices to detect the attack, such as the wavelength quantum filter to filter invisible photons and the photon number splitter(PNS) to discover the delay photons. If the multi-photon rate is unreasonable high, then such attack can be detected.

#### Fairness Analysis

The dishonest participants pose a greater threat to the security of the protocol than outside eavesdroppers. As we mentioned above, the dishonest participant can take the advantage position or collaborate with others to predetermine the final key. Following we conduct a fairness analysis to show that our protocol can resist participant’s attacks.

Let’s discuss the first attack strategy. For the sake of convenience, we suppose there are only three participants Alice, Bob and Charlie, wherein Bob is dishonest. In step (5), Bob selects an advantageous geographical position aside Alice and Charlie so he can obtain Alice’s and Charlie’s controlling sequence *RH*_*i*_^*j*^ earlier than expected. According to the controlling sequences, Bob can perform the operations ${R}_{z}(\frac{-{C}_{i}^{j}\pi }{2})$ to remove the additional controlling operations and perform Bell measurements to obtain the final key in advance. Then Bob wants to predetermine the final keys of Alice and Charlie by announcing incorrect controlling sequences to them. However, we request that each participant first announces the controlling sequence before they cooperate to choose photons for security checking, so this ineluctably leads to the photon pairs for security checking in DBS basis being measured in BS basis and collapsing randomly into one of the four Bell states. Suppose the number of final keys which Bob wants to change is *m*, there is a $\frac{kl}{l+kl}$ probability that the selected photons are for security checking since bob cannot unambiguously distinguish the photons for security checking and for final keys. The probability that Bob will successfully pass the security checking is ${(\frac{1}{2})}^{\frac{klm}{l+kl}}={(\frac{1}{2})}^{\frac{km}{1+k}}\approx 0$ and predetermine the final key is ${(\frac{1}{2})}^{\frac{lm}{l+kl}}={(\frac{1}{2})}^{\frac{m}{1+k}}\approx 0$ according to Table 2 (if the number *m* is large enough). So the dishonest participant cannot predetermine the final keys of honest participants and the protocol can achieve fairness property.

Following we analyze the collusive attack. The worst case is that only one participant is honest and all others are dishonest. Let’s take three participants *P*_1_, *P*_2_ and *P*_3_ for example, where *P*_1_ and *P*_3_ are dishonest. They want to predetermine *P*_2_’s final key. The detailed attack strategies are as follows. *P*_1_ prepares Bell states and sends the photon sequence |*BS*_2_^1→2^〉 to *P*_2_. After *P*_2_ completes his operations on the photon sequence |*BS*_2_^1→2^〉 and sends the sequence |*BS*_2_^1→3^〉 to *P*_3_, *P*_1_ and *P*_3_ won’t measure the Bell states until step (5) where each participant publishes their additional controlling sequences. After obtaining *P*_2_’s controlling sequence *RH*_2_^1^, *P*_1_ and *P*_3_ can deduce *P*_2_’s secret key *K*_2_. However, the only method for *P*_1_ and *P*_3_ to determine the final key of *P*_2_ is to announce fake controlling sequences to him. Based on the analysis of the first participant’s attack strategy, we can conclude the probability they will successfully pass the security checking and predetermine *P*_2_’s final key is close to 0. Therefore *n*−1 dishonest participants cannot determine the final key. In summary, our proposed protocol can resist participant’s attacks.

#### Information leakage analysis

In addition to the above attacks, information leakage should also be considered. In our protocol, only the controlling string *RH*_*i*_^*j*^ needs to be published in stage (5). Since *RH*_*i*_^*j*^ has nothing to do with the secret key, Eve can only guess that the operation performed by each participant is either I or Z, which contains $-2\times \frac{1}{2}{\log }_{2}\frac{1}{2}=1$ bit of uncertain information for Eve. As a result, Eve cannot obtain any information of secret key without taking any active attacks. The problem of information leakage does not exist in our agreement.

### Efficiency analysis

Following we compare the proposed MQKA protocol with the existing four secure protocols, i.e., LGHW13 protocol^20^, HSXL16 protocol^33^, CM17 protocol^34^ and HSL17 protocol^32^, in five aspects: qubit efficiency *η*_*q*_, measurement efficiency *η*_*m*_, unitary operation efficiency *η*_*u*_, quantum resource and category of the protocol. The definitions are as follows: qubit efficiency *η*_*q*_ = $\frac{l}{q}$, measurement efficiency *η*_*m*_ = $\frac{l}{m}$, and unitary operation efficiency *η*_*u*_ = $\frac{l}{u}$, where *l* denotes the length of the final common key, *q* is the number of the transmitted qubits on the quantum channel, *m* is the number of quantum measurements, and *u* is the number of unitary operations. Table 3 shows the detailed comparison results between these four MQKA protocols and ours. The efficiency analysis is given as follows.

**Table 3:** Comparison between existing security protocols.

Protocol	*η*_*q*_	*η*_*m*_	*η*_*u*_	Quantum resource	Category
LGHW13	$\frac{1}{n(n-\mathrm{1)}(1+k)}$	$\frac{1}{n(n-\mathrm{1)}(1+k)}$	0	Single photons	Complete-graph
HSXL16	$\frac{1}{n\mathrm{(1}+kn)}$	$\frac{1}{n\mathrm{(1}+kn)}$	$\frac{1}{{n}^{2}}$	Single photons	Circle
CM17	$\frac{1}{n\mathrm{(1}+kn)}$	$\frac{1}{n\mathrm{(1}+kn)}$	$\frac{2}{n(n+\mathrm{1)}}$	Two particles	Circle
*HSL*17	$\frac{1}{n\mathrm{(1}+k)}$	$\frac{1}{n\mathrm{(1}+k)}$	$\frac{1}{{n}^{2}\mathrm{(1}+k)}$	Single photons	Circle
Ours	$\frac{1}{n\mathrm{(1}+k)}$	$\frac{1}{n(1+\frac{k}{n})}$	$\frac{1}{{n}^{2}\mathrm{(1}+k)}$	Bell states	Circle

*η*_*q*_, *η*_*m*_ and *η*_*u*_ are qubit efficiency, measurement efficiency and unitary operation efficiency, respectively.

In our protocol, each participant will prepare *l* + *kl* photon pairs to establish *l*-bit final key, wherein *kl* bits are used for security detection. As there are only half photon sequence transmitted in the quantum channel and *n* participants involved in our protocol, the total number of transmitted photons on the quantum channel is *n*(*l* + *kl*). Hence, the qubit efficiency is

5 $$\frac{l}{n(l+kl)}=\frac{1}{n\mathrm{(1}+k)}\mathrm{.}$$

Since only one eavesdropping detection for each participant, the number of measurements required in this protocol is greatly reduced. To establish an *l*-bit final key, each participant needs to perform $l+\frac{kl}{n}$ measurements. Therefore, the measurement efficiency of our protocol is

6 $$\frac{l}{n(l+\frac{kl}{n})}=\frac{1}{n(1+\frac{k}{n})}.$$

The security of our protocol is mainly based on the controlling operations of each participant on the photon sequences. To establish an *l*-bit final key, each participant needs perform *n*(*l* + *kl*) unitary operations. Thus, the unitary operation efficiency of the proposed protocol is

7 $$\frac{l}{{n}^{2}(l+kl)}=\frac{1}{{n}^{2}\mathrm{(1}+k)}\mathrm{.}$$

**Figure 2:**
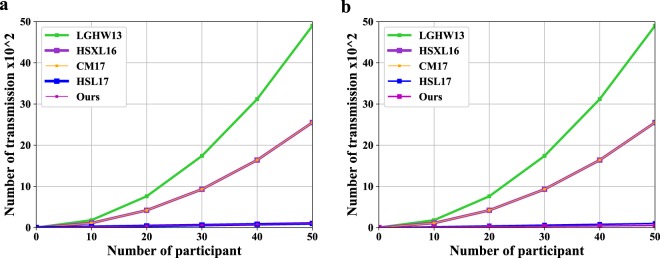
The comparisons of the number of transmissions and measurements, where k = 1.

The specific comparison results are shown in Fig. 2. As shown in the two subgraphs (a) and (b), the qubit efficiency of the improved protocol is no less than that of the existing security protocols, and it has higher measurement efficiency. Although we increase the number of unitary operations in exchange for higher qubit efficiency and measurement efficiency, the unitary operations can be easily realized with the rapid development of quantum technology. Therefore our protocol is efficient and feasible.

### Optical setup

**Figure 3:**
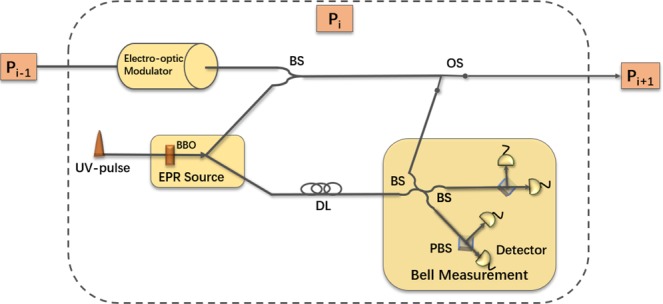
Experimental setup of participants. BBO: beta barium borate. BS: beam splitter. OS: optical switch. DL: delay line. PBS: polarization beam splitter. Each participant can generate and measure the polarization-entangled photon pairs, encode the received photon sequence and send the photon sequence to next participant.

As shown in Fig. 3, we design an optical setup for each participant. In the experiment, ultraviolet (UV) laser pulses pass through a BBO crystal to produce polarization-entangled photon pairs^42^. One of the photon pairs can be first stored in *P*_*i*_c delay line and the other is sent to *P*_*i*+1_. *P*_*i*_ encodes his secret key and controlling information on other participant’s photon sequence by utilizing electro-optic modulator^43^ and sends the photon sequence to next participant *P*_*i*+1_. This process continues until *P*_*i*_ receives the sequence which he generates. After offsetting the extra controlling operations on his second photon sequence by utilizing electro-optic modulator, *P*_*i*_ fetches the first photon sequence from the delay line and performs Bell measurement^42^ on the photon pairs. According to the measurement results and initial states, all participants can obtain the consistent final key.

## Conclusion

In this paper, we find that some existing travelling-mode MQKA protocols are generally vulnerable to the internal dishonest participants. Besides, we also find the problem of information leakage in Cao-Ma MQKA protocol. Then We take Cao-Ma MQKA protocols as examples to illustrate these attacks in detail. To resist the attacks, we propose a robust travelling-mode MQKA protocol based on non-orthogonal Bell states. The analyses show that our protocol can resist the both outside and participant’s attacks and achieve higher efficiency. Finally, We design an optical platform for each participant, and show that our proposed protocol can be realized with feasible technologies.
